# High Genetic Diversity and Different Distributions of Glycosyl Hydrolase Family 10 and 11 Xylanases in the Goat Rumen

**DOI:** 10.1371/journal.pone.0016731

**Published:** 2011-02-03

**Authors:** Guozeng Wang, Huiying Luo, Kun Meng, Yaru Wang, Huoqing Huang, Pengjun Shi, Xia Pan, Peilong Yang, Qiyu Diao, Hongfu Zhang, Bin Yao

**Affiliations:** 1 Key Laboratory for Feed Biotechnology of the Ministry of Agriculture, Feed Research Institute, Chinese Academy of Agricultural Sciences, Beijing, People's Republic of China; 2 State Key Lab of Animal Nutrition, Institute of Animal Science, Chinese Academy of Agricultural Sciences, Beijing, People's Republic of China; New England Biolabs, Inc., United States of America

## Abstract

**Background:**

The rumen harbors a complex microbial ecosystem for efficient hydrolysis of plant polysaccharides which are the main constituent of the diet. Xylanase is crucial for hemicellulose hydrolysis and plays an important role in the plant cell wall degradation. Xylanases of ruminal strains were widely studied, but few studies have focused on their diversity in rumen microenvironment.

**Methodology/Principal Findings:**

We explored the genetic diversity of xylanases belonging to two major glycosyl hydrolase families (GH 10 and 11) in goat rumen contents by analyzing the amplicons generated with two degenerate primer sets. Fifty-two distinct GH 10 and 35 GH 11 xylanase gene fragments (similarity <95%) were retrieved, and most had low identities with known sequences. Based on phylogenetic analysis, all GH 10 xylanase sequences fell into seven clusters, and 88.5% of them were related to xylanases from Bacteroidetes. Five clusters of GH 11 xylanase sequences were identified. Of these, 85.7% were related to xylanases from Firmicutes, and 14.3% were related to those of rumen fungi. Two full-length xylanase genes (one for each family) were directly cloned and expressed in *Escherichia coli*. Both the recombinant enzymes showed substantial xylanase activity, and were purified and characterized. Combined with the results of sheep rumen, Bacteroidetes and Firmicutes are the two major phyla of xylan-degrading microorganisms in rumen, which is distinct from the representatives of other environments such as soil and termite hindgut, suggesting that xylan-degrading microorganisms are environment specific.

**Conclusion/Significance:**

The numerous new xylanase genes suggested the functional diversity of xylanase in the rumen microenvironment which may have great potential applications in industry and agriculture. The phylogenetic diversity and different distributions of xylanase genes will help us understand their roles in plant cell wall degradation in the rumen microenvironment.

## Introduction

Plant cell walls mainly consist of cellulose, hemicelluloses, and lignin, and are a major component of the diet of grazing ruminants [1]. The rumen ecosystem consists of various anaerobic microorganisms including bacteria, fungi, protozoa, and archaea that are characterized by high population density, wide diversity, and complexity of interactions [2]. These rumen microbes work synergistically to efficiently hydrolyze plant cell wall polysaccharides by producing various enzymes, such as cellulases, hemicellulases, polyphenol oxidases, esterases, etc [2], [3], [4].

Hydrolysis of the plant cell wall is a complex process in which hemicellulose digestion is the initial step with subsequent hydrolysis of the cellulose [5], [6]. Xylan is the major component of hemicellulose, and its complete hydrolysis requires a crucial enzyme—endo-1,4-β-d-xylanase (EC 3.2.1.8)—to cleave xylan into short xylooligosaccharides of varying lengths [7], [8]. Xylanases have been found in rumen bacteria, fungi, and protozoa but not yet archaea [2], and some xylanases display unique structures and characteristics [9]. Using protein purification and molecular cloning methods, researchers have detected multiple xylanases in rumen bacteria and fungi [10], [11], [12]. Moreover, whole-genome sequencing data of rumen bacteria [13] (http://www.jcvi.org/rumenomics/) further provide a more comprehensive understanding of the diversity of xylanase genes, their distribution in various bacteria, and their roles in degrading plant cell wall polysaccharides [14], [15], [16].

The genetic diversity of rumen microbial communities based on 16S rDNA sequences has been widely studied, and results have suggested a high diversity of rumen microbes with the majority of them not yet cultured [17]. Rumen metagenomics have revealed the diversity of glycosyl hydrolases (GH) of rumen microbes [3], [18], but far fewer xylanase genes have been obtained using these methods relative to the large number of uncultured microorganisms in the rumen ecosystem. Culture-independent study of functional gene diversity has allowed access to the uncultured rumen microenvironment and provided insight into metabolic capabilities of uncultivated microbial communities [19]. Moreover, xylanases from these uncultured microbes may be of special interest because of their functional diversity and potential applications [18], [19].

The objective of this study was to explore the genetic diversity of xylanases in goat rumen using culture-independent molecular approaches. Sequence analysis showed that most of the xylanase gene fragments have low identities to known xylanases, suggesting the existence of a large number of uncharacterized xylanase genes in rumen. To confirm that these genes encode active enzymes, one GH 10 and one GH 11 full-length xylanase genes were directly cloned from the rumen genomic DNA and expressed in *Escherichia coli*. One of the xylanases showed high activity even at relatively low temperatures. Our study provides new insight into the genetic diversity and distribution of xylanases, which will help us understand their roles in the rumen microenvironment.

## Results

### Amplification and sequence analysis of GH 10 xylanase gene fragments

Using degenerate primers X_10_-F and X_10_-R [20], gene fragments of about 260 bp for GH 10 xylanases were amplified directly from the total genomic DNA of goat rumen contents. A clone library was constructed, and positive transformants (white colonies) were confirmed by PCR with primers M13F and M13R. Of the 310 clones sequenced, 236 sequences showed 35–84% amino acid identity with known GH 10 xylanases based on BLAST analysis, and no fragment contained introns. The highly conserved Asn residue of GH 10 xylanases [21] was identified in all of these protein sequences. The result suggested that these sequences were partial xylanases and that some of them might be previously unidentified.

After removing the redundant sequences using CD-hit program [22], 52 sequences showed divergence (sharing <95% identity) (Table S1). The length of these sequences varied a lot, with the range from 84 to 111 residues. Abundance analysis using distance-based operational taxonomic unit and richness determination (DOTUR) software [23] showed that GR117 was the predominant operational taxonomic unit (OUT) that represented 30 sequences. Twelve OTUs contained only one sequence (Table S1).

### Phylogenetic analysis of GH 10 xylanase gene fragments

**Figure 1:**
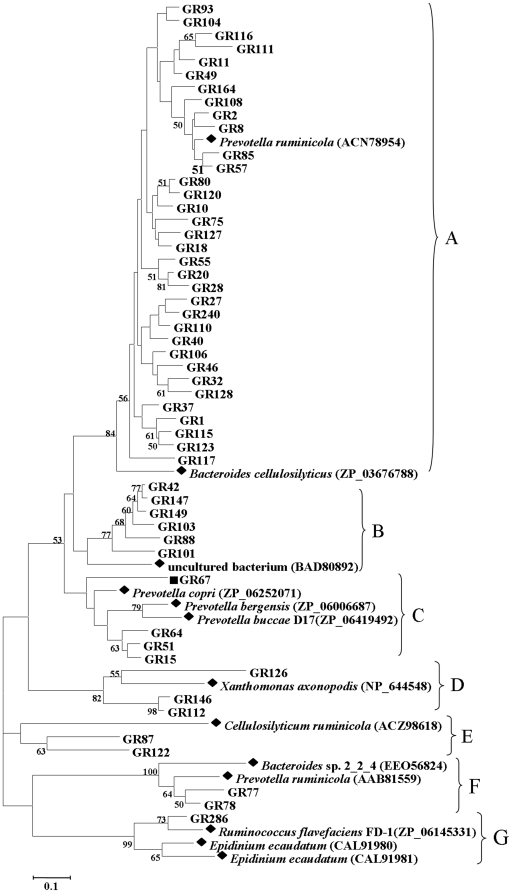
Phylogenetic analysis based on the partial amino acid sequences of GH 10 xylanase genes detected in the goat rumen contents and their relationship with the reference sequences retrieved from GenBank. This tree was constructed using the neighbor-joining method (MEGA 4.0). The lengths of the branches indicate the relative divergence among the amino acid sequences. The reference sequences are marked with a closed diamond (♦) with source strains and GenBank accession numbers in parentheses. The gene fragments (GR67) used for full-length cloning were marked with a solid square (▪). The numbers at the nodes indicate bootstrap values based on 1,000 bootstrap replications and bootstrap values (>50) are displayed. The scale bar represents 0.1 amino acid substitutions per position.

An unrooted protein-level phylogenetic tree for GH 10 xylanases was constructed with the 52 divergent sequences from the GH 10 clone library and 13 reference sequences from GenBank. All the sequences were confined to seven clusters, denoted A, B, C, D, E, F, and G, indicating substantial diversity among GH 10 xylanases in goat rumen (Figure 1). Many sequences had no close relatives.

Cluster A contained 34 sequences from the goat rumen and two reference sequences from *Prevotella ruminicola* and *Bacteroides cellulosilyticus*. Cluster B contained the sequences of six clones and one xylanase directly cloned from an uncultured human gut bacterium. This reference sequence was highly similar to xylanases from anaerobic intestinal bacteria such as *Bacteroides* spp. and *Prevotella* spp. [24]. Four sequences as well as three reference sequences from *Prevotella buccae*, *Prevotella bergensis*, and *Prevotella copri* formed cluster C. Three sequences in cluster D were closely related to the xylanase from *Xanthomonas axonopodis*. In cluster E, there were two sequences that shared highest identity with the xylanase from *Cellulosilyticum ruminicola*. Two sequences in cluster F were closely related to the xylanases from *P. ruminicola* and *Bacteroides* sp. 2_2_4. Cluster G contained one sequence from our library and two reference sequences from *Ruminococcus flavefaciens* FD-1 and *Epidinium ecaudatum.*

### Amplification and sequence analysis of GH 11 xylanase gene fragments

Amplicons with the size of about 210 bp for GH 11 xylanases were obtained from the metagenomic DNA of goat rumen contents using degenerate primers X_11_-F and X_11_-R [20]. Of the clone library constructed with PCR product recovered, 200 clones were randomly sequenced, and 172 sequences showed 56–95% amino acid identity with known GH 11 xylanases. The highly conserved catalytic residue, Glu, of GH 11 xylanases [25], [26] was found in all the protein sequences of 70–81 residues in length (Table S2). Thus these sequences were identified to be partial GH 11 xylanases, and some of them were yet undiscovered.

After removing the redundant sequences using CD-hit program [22], 35 sequences showed divergence (sharing <95% identity) (Table S2). DOTUR abundance analysis showed that R8 was the predominant OTU that represented 15 sequences. Four OTUs contained only one sequence (Table S2).

### Phylogenetic analysis of GH 11 xylanase gene fragments

**Figure 2:**
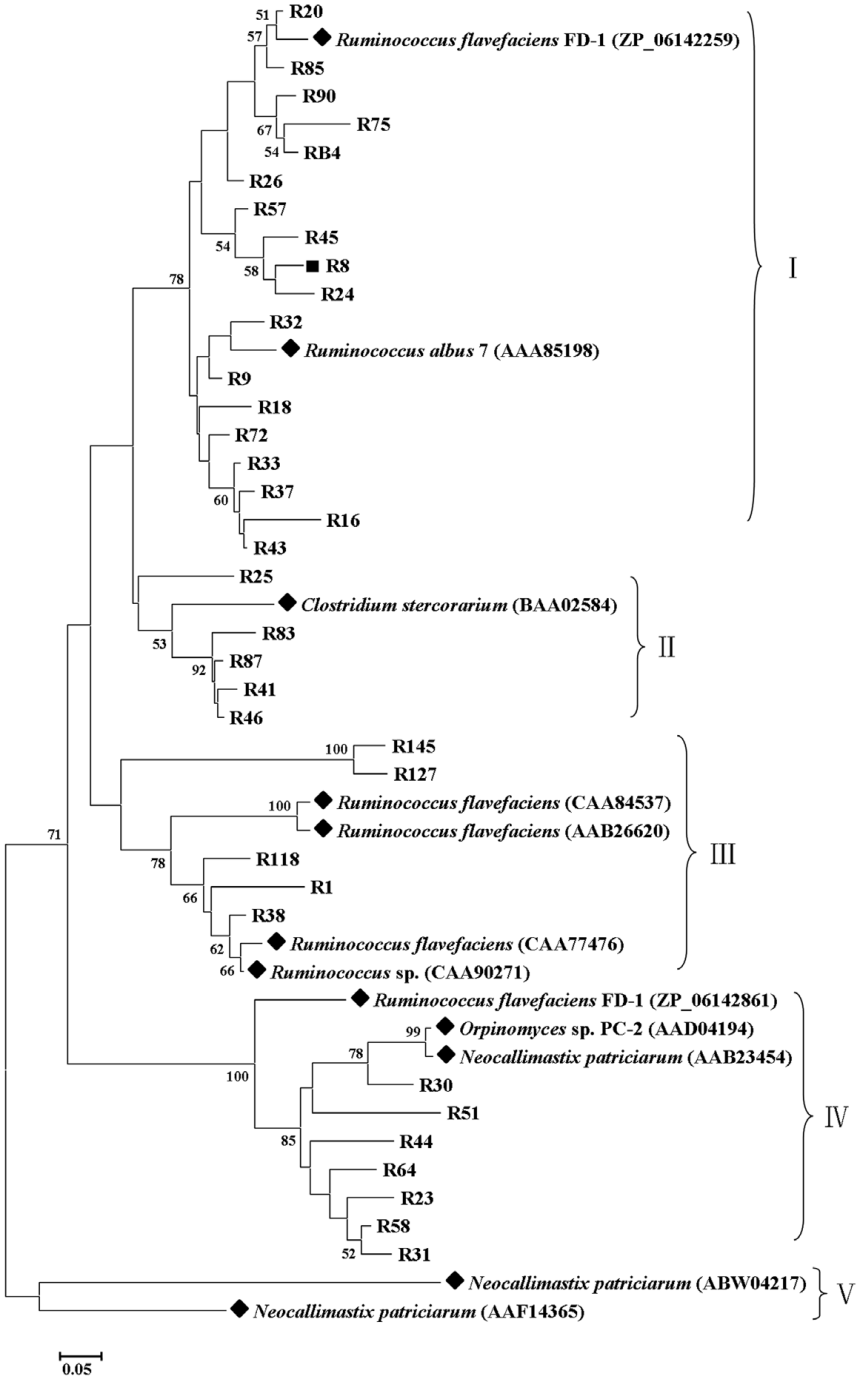
Phylogenetic analysis based on the partial amino acid sequences of GH 11 xylanase genes detected in the goat rumen contents and their relationship with the reference sequences retrieved from GenBank. This tree was constructed using the neighbor-joining method (MEGA 4.0). The lengths of the branches indicate the relative divergence among the amino acid sequences. The reference sequences are marked with a closed diamond (♦) with source strains and GenBank accession numbers in parentheses. The gene fragment (R8) used for full-length retrieving is marked with a solid square (▪). The numbers at the nodes indicate bootstrap values based on 1,000 bootstrap replications and bootstrap values (>50) are displayed. The scale bar represents 0.05 amino acid substitutions per position.

The 35 distinct partial sequences of GH 11 xylanases were used to construct an unrooted phylogenetic tree with 12 reference sequences (Figure 2). Five clusters (I, II, III, IV, and V) were formed based on high bootstrap values. Many clades formed without closely related references, suggesting that these sequences were probably different from known xylanases. Twenty-two sequences shared highest identity with xylanases from *Ruminococcus albus*, *R. flavefaciens*, and *Ruminococcus* sp., and fell into clusters I and III. Five sequences and that of *Clostridium stercorarium* were grouped into cluster II. Seven sequences closely related to xylanases from *Neocallimastix patriciarum*, *Orpinomyces* sp. PC-2, and *R. flavefaciens* fell into cluster IV. Cluster V only contained two references from *N. patriciarum.*

### Cloning and expression of xylanase genes

Two full-length xylanase genes were directly cloned from the metagenomic DNA of goat rumen contents. The complete sequence of *xynGR67* contained an open reading frame of 1,239 bp encoding a 412-residue polypeptide with a typical signal peptide (residues 1–40). Sequence similarity searches showed that deduced XynGR67 shared highest identity (45%) with the GH 10 xylanase from Flavobacteriaceae bacterium 3519-10, an isolate recovered from a deep Antarctic ice core [27].

The complete sequence of *xynR8* contained an open reading frame of 807 bp that encoded a 268-residue polypeptide. No signal peptide was predicted. The deduced protein shared highest identity with the GH 11 xylanases from *R. flavefaciens* FD-1 [14] and *R. albus* 8 (75% and 64%, respectively).

Both genes encoding the mature proteins were expressed in *E. coli* BL21 (DE3). After induction with IPTG at 25°C for 12 h, substantial xylanase activity was detected in the culture supernatant of recombinant cells (11.6 and 27.6 U ml^–1^ for XynGR67 and XynR8, respectively).

### Biochemical characterization of purified recombinant XynGR67 and XynR8

**Figure 3:**
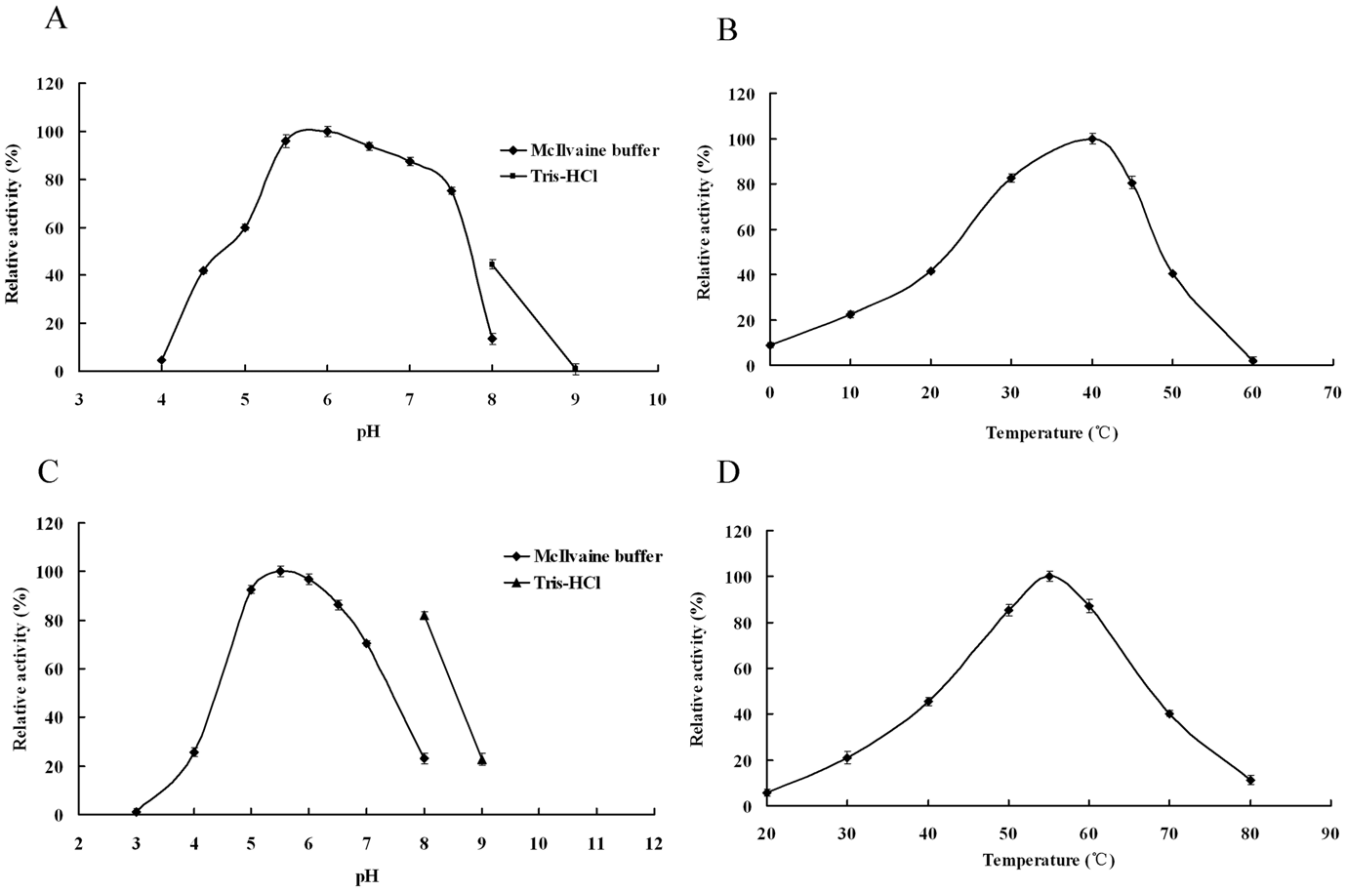
pH and temperature activity profiles of purified recombinant XynGR67 and XynR8. **A** Effect of pH on XynGR67 activity. Activities at various pHs were assayed at 30°C. **B** Effect of temperature on XynGR67 activity in McIlvaine buffer (pH 6.0). **C** Effect of pH on XynR8 activity. Activities at various pHs were assayed at 30°C. **D** Effect of temperature on XynR8 activity in McIlvaine buffer (pH 5.5). The error bars represent the mean ± SD (n = 3).

Using birchwood xylan as the substrate, XynGR67 showed the highest activity at pH 6.0, and >75% of the maximum activity was retained at pH 5.5–7.5 (Figure 3A). The enzyme was stable at pH 5.0–8.0, retaining more than 80% of the initial activity after incubation at 25°C for 1 h. The optimal temperature for enzyme activity of XynGR67 was 40°C (Figure 3B). At 0°C and 10°C, the enzyme still exhibited 8.2% and 22.5% of the maximal activity, respectively. XynGR67 was very thermolabile, retaining 65% of the activity after 1 h incubation at 30°C, and losing activity rapidly when incubated at 40°C (a half-life of 15 min).

XynR8 showed the highest activity at pH 5.5, and >80% of the maximum activity was retained at pH 5.0–8.0 (Figure 3C). The enzyme was stable at pH 5.0–9.0, retaining more than 80% of the initial activity after incubation at 37°C for 1 h. The optimal temperature for enzyme activity of XynR8 was 55°C (Figure 3D). XynR8 was thermostable, retaining 85% of the activity after 1 h incubation at 50°C, and remaining 31% activity when incubated at 60°C for 1 h.

Kinetic parameters of purified recombinant XynGR67 and XynR8 on birchwood xylan were shown in Table 1. Both enzymes showed substantial xylanase activity to birchwood xylan. The substrate specificity of XynGR67 and XynR8 were shown in Table 2. Of four types of xylan tested, XynGR67 had the highest activity toward beechwood xylan while XynR8 displayed the highest activity toward soluble wheat arabinoxylan.

**Table 1:** Kinetic parameters of XynGR67 and XynR8 towards birchwood xylan.

Enzyme	Temperature (°C)	*V*_max_(µmol min^–1^ mg^–1^)	*K*_m_(mg ml^–1^)	*k_cat_*(s^–1^)	*k*_cat_/*K*_m_(ml s^–1^ mg^–1^)
XynGR67	40	2,150.0±72.4	1.43±0.16	1,505.0±50.4	1,052.5
	4	500.4±14.3	1.96±0.17	350.0±10.1	178.6
XynR8	55	2,647.9±60.9	3.27±0.22	1,306.3±30.1	399.5

**Table 2:** Substrate specificity of the purified recombinant XynGR67 and XynR8.

Substrate	Relative activity (%)	
	XynGR67	XynR8
Birchwood xylan	100	100
Beechwood xylan	150.7±3.1	196.8±2.7
Wheat arabinoxylan	53.4±2.5	384.4±3.4
Wheat arabinoxylan (insoluble)	63.1±1.9	54.6±2.2
pNP-cellobioside	NA	NA
pNP-xyloside	NA	NA

## Discussion

In this study, xylanase genes were targeted for diversity analysis because of their key roles in the initial steps of plant cell wall breakdown and their great potential for industrial and agricultural applications [7], [8], [28]. The microorganisms in the rumen are immensely diverse, and the microbial-mediated hydrolysis of plant cell wall polysaccharides is highly efficient [2]. Thus, the rumen is an ideal microenvironment to study the genetic diversity of functional xylanases.

Application of degenerate primers designed based on CODEHOP principles [20] resulted in the discovery of 52 distinct GH 10 and 35 GH 11 xylanase gene fragments from goat rumen contents. Most sequences had low identities with known xylanases in GenBank, implying that these xylanases may be as yet undiscovered. Moreover, these amplified partial sequences had low similarities and were distantly related based on phylogenetic analysis (Figures 1 and 2). These results suggest that there are a large number of unidentified xylanase genes in rumen, corresponding to the large number of uncultured microorganisms in the rumen. Compared with previous culture-independent methods such as metagenomic library construction and screening [18], pyrosequenced rumen metagenome [3], and termite hindgut metagenomics [29], far more xylanase genes were documented in our present study, and they were more diverse (Table 3), suggesting that the PCR-based culture-independent method is an efficient way to identify xylanase genes in the rumen.

**Table 3:** Amount of GH 10 and 11 xylanases in the genomes of *Ruminococcus flavefaciens* FD-1, *Prevotella ruminicola* 23, pyrosequenced rumen, termite hindgut and goat rumen in this study.

Source	GH 10	GH 11	References
*Ruminococcus flavefaciens* FD-1 (Rf)	6	11	2
*Ruminococcus albus* 7	5	6	GenBank database
*Fibrobacter succinogenes* S85	2	3	GenBank database
*Prevotella ruminicola* 23	2	0	GenBank database
*Prevotella bryantii* B14	2	0	GenBank database
Pooled liquid	10	2	6
Fiber-8	5	ND	6
Fiber-64	7	1	6
Fiber-71	4	ND	6
Termite hindgut	46	14	46
**Goat rumen**	52	35	This study

Rumen bacteria and fungi are the key agents to degrade plant fiber [2], [30]. It has been proposed that rumen fungi play an important role in the initiation of plant fiber degradation during fermentative digestion in ruminants [2], [12]. Pyrosequenced rumen metagenome analysis by Brulc *et al.* [3] showed that all the xylanases they examined were bacterial, and no sequence was closely related to those of fungi. In our present study, however, seven GH 11 xylanase gene fragments were retrieved directly from the rumen and showed a close relationship with rumen fungi based on phylogenetic analysis (Figure 2). Among these seven xylanase sequences, five had the highest identity to xylanases from *N. patriciarum* and *Orpinomyces* sp. PC-2, and two were closely related to those from *R. flavefaciens* FD-1 (Table S2). This result suggests that horizontal gene transfer from rumen bacteria to fungi may have occurred [31], [32]. In addition, the distribution of GH 11 xylanases in rumen differed from that in both tundra soil (in which more fungal than bacterial xylanase genes were detected) [20] and termite hindgut (in which GH 11 xylanases were mainly from the phyla Fibrobacteres and Spirochaetes) [29]. This result suggests that the rumen microenvironment differs from other ecosystems and has specific xylan-degrading microbial community.

Firmicutes is one of the two most abundant bacteria in rumen based on 16S rDNA library-based analysis [2], [17]. The representative genus of xylan-degrading Firmicutes in rumen is *Ruminococcus* [14]. Our current study showed that 33 distinct sequences (30 of GH 11 and 3 of GH 10) are closely related to the xylanases from Firmicutes, which comprises 38% of the total distinct sequences. Among them, 25 sequences (24 of GH 11 and 1 of GH 10) are related to *Ruminococcus*. Abundance analysis also showed that xylanase genes related to *Ruminococcus* are dominant in goat rumen (Table S2). The OTU representative of GH 11 xylanase gene fragments, R8, was subjected to full-length gene cloning and heterogeneous expression. The recombinant enzyme showed high activity to xylan. All these results suggest that the genus *Ruminococcus* plays an important role in xylan degradation in the goat rumen.

The Cytophaga-Flexibacter-Bacteroides group represents another abundant group of bacteria in rumen [2], [17]. Based on phylogenetic analysis, 46 GH 10 xylanase sequences were closely related to the xylanases from Bacteroides, comprising 53% of the total distinct sequences. Abundance analysis also showed that xylanase genes related to Bacteroides were dominant in goat rumen (Table S1). More than half of them (26/46) were closely related to the xylanases from *Prevotella*, which is one of the most important xylan-degrading bacterial genera in rumen [33], [34]. Many new clades were formed despite a lack of relatives, suggesting that there are other unidentified xylanase-producing Bacteroides in goat rumen. In addition to xylanases from Bacteroides, xylanase fragments from other phyla were also detected in the goat rumen. Three sequences (GR112, GR126, and GR146) in the GH 10 clone library had the highest similarity with xylanases from *X. axonopodis* and Verrucomicrobiae bacterium. This is the first report of xylanase genes obtained from rumen that is homologous to Verrucomicrobia and Proteobacteria xylanase.

In general, rumen microorganisms can be classified into two major groups: cellulolytic and noncellulolytic [2]. The cellulolytic microorganisms include cellulolytic rumen bacteria, fungi, and some protozoa that can utilize both cellulose and xylan as a carbon source. These microorganisms, such as *Fibrobacter succinogenes*, *R. flavefaciens*, and *Neocallimastix frontalis*, express both GH 10 and GH 11 xylanases [12], [15, [16]. The noncellulolytic microorganisms include members of the dominant Gram-negative Cytophaga-Flexibacter-Bacteroides phylum and Gram-positive Firmicutes, such as *Butyrivibrio fibrisolvens*, *Roseburia* sp., and *Eubacterium rectale*, which can use soluble products resulting from plant cell wall breakdown [2]. These rumen microorganisms produce only GH 10 xylanases. In our study, about 88.5% (46/52) of the GH 10 xylanase sequences were closely related to Bacteroides xylanases, whereas 85% (30/35) of the GH 11 xylanase sequences were related to xylanases from cellulolytic Firmicutes, confirming the primary distribution of GH 10 xylanase genes in noncellulolytic microorganisms and GH 11 xylanase genes in cellulolytic-xylanolytic microorganisms. GH 10 and GH 11 xylanases are distinct from each other in both three-dimensional structure [35] and mechanism of action [25]. The products of GH 11 xylanases can be further hydrolyzed by GH 10 enzymes [7]. Therefore, the different distributions of these two families imply their different roles in xylan degradation in the rumen.

To date, only a few xylanase genes have been obtained from uncultured microorganisms in rumen by direct cloning [36] or screening from the metagenomic library of rumen [18]. In our current study, two full-length xylanase genes were directly obtained from the metagenomic DNA of goat rumen contents. Both enzymes showed high activity towards different natural xylans and displayed different characteristics (Tables 1 and 2). XynGR67 had low identity (45% at maximum) with known xylanases in GenBank and similarity to a characterized cold-active xylanase [37]. Although the optimum temperature of XynGR67 was 40°C, substantial activity remained at low temperatures (22.5% at 10°C and 8.2% at 0°C). The enzyme was also thermolabile, showing a half-life of 15 min at 40°C. Compared with the cold-active xylanase XynA from *Glaciecola mesophila* [37], XynGR67 displayed a much higher *k*_cat_/*K*_m_ value (178.5 vs. 15.4) at 4°C. These data suggest that XynGR67 has some cold-active characteristics and is distinct from other known ruminal xylanases.

Using molecular methods, bacterial composition in the gastrointestinal tract are found to be dependent on host, diet, age and so on [38], [39], [40]. Of these factors, host species is the most important one [41], [42]. To expand our assessment of the xylanase gene diversity in rumen microevironment, the rumen contents of a Small Tail Han sheep that grazed under the same conditions as the Boer goat under study were subjected to xylanase gene analysis (Tables S3 and S4). Both rumens harbor similar xylan-degrading communities based on the sequence comparison analysis (Table S5, Figures S1 and S2). Similar or identical sequences were found in both rumens, and the vast majority of the amplified xylanase fragments belonged to the two major phyla of Firmicutes and Bacteroides. Based on UniFrac analysis [43], the GH 10 xylanase gene fragments in both rumens were marginally different (P = 0.02) while there was no significant difference in that of GH 11 (P = 0.35). Although the abundant sequences in each rumen are different (Table S5), the predominant OTUs of different rumens shared high identities (68% for GH 10 and 89% for GH 11, respectively). A small part of sequences are unique in each rumen (Figures S2), implying that some xylan-degrading microorganisms might be host specific [41]. The strategy reported here can be applied to explore other gastrointestinal tract ecosystems known to be highly specialized for raw biomass degradation (i.e., human and insect gut microbiomes) for novel xylanases.

In conclusion, an efficient culture-independent molecular method was used to explore the diversity of xylanases in the rumen ecosystem. Sequence analysis revealed a large number of unidentified and potential new xylanases in the goat rumen. Full-length cloning and heterologous expression of some genes further confirmed their function as active xylanases. The majority of GH 10 xylanase sequences were from noncellulolytic microorganisms, whereas most of the GH 11 sequences were from cellulolytic microorganisms, implying their different roles in xylan degradation in the rumen ecosystem. Moreover, xylanase distribution in different environments is variable, suggesting that xylan-degrading microorganisms are environment specific.

## Materials and Methods

### Ethics statement

All animal studies were followed the regulation for the review committee of laboratory animal welfare and ethics and protocol for the review on laboratory animal welfare and ethics, Beijing Administration Office of Laboratory Animal. The animal experimentation was approved by the Committee of Laboratory Animal Welfare and Ethics, Beijing Administration Office of Laboratory Animal with the approval No. SYXK2008-0007.

### Sample collection and DNA extraction

A three-year-old male Boer goat that had grazed on late-fall pasture in Inner Mongolia, China was selected. Total rumen contents were collected immediately after slaughtering and stored at −70°C. The biomass was collected by centrifuging at 17,000×*g*, 4°C for 30 min. The sample used for DNA extraction were frozen in liquid nitrogen and ground to a fine powder with a mortar. Total genomic DNA was extracted following a protocol specific for high molecular weight DNA from environmental samples [44] and purified with an Agarose Gel DNA Purification kit (TaKaRa, Japan). The final DNA concentration was ∼80 ng µl^–1^.

### PCR amplification of xylanase gene fragments

Two degenerate primer sets (X_10_-F: 5′-CTACGACTGGGAYGTNIBSAAYGA-3′ and X_10_-R: 5′-GTGACTCTGGAWRCCIABNCCRT-3′; and X11-F: 5′-AACTGCTACCTGKCNITNTAYGGNTGG-3′ and X11-R: 5′-CCGCACGGACCAGTAYTGNKIRAANGT-3′) specific for GH 10 and GH 11 xylanases, respectively, were used to amplify xylanase gene fragments with the purified DNA as a template following the reaction system and PCR conditions as reported [20]. PCR products were visualized on an agarose gel, and bands of the expected size (∼260 bp for GH 10 and 210 bp for GH 11 xylanases) were excised and purified with the TaKaRa Agarose Gel DNA Purification kit.

### Cloning and sequencing of PCR products

To construct the clone library for each xylanase family, the purified PCR products were ligated into vector pGEM-T Easy (Promega, USA) and electroporated into *E. coli* DH5α competent cells (TaKaRa). Cells were grown on Luria-Bertani agar plates containing 100 µg ml^−1^ ampicillin, 80 µg ml^−1^ X-Gal, and 0.5 µM isopropyl-β-d-1-thiogalactopyranoside (IPTG) at 37°C for 15 h. The positive transformants (white clones) in each library were randomly picked, amplified with primers M13F (5′-GTAAAACGACGGCCAGT-3′) and M13R (5′-GGATAACAATTTCACACAGGA-3′), and sequenced by Sunbiotech (China) for confirmation.

### Phylogenetic analysis

Nucleotide sequences of the xylanase gene fragments were translated into amino acid sequences with EMBOSS Transeq (http://www.ebi.ac.uk/emboss/transeq) and aligned at the protein level with known sequences in the GenBank database with ClustalW. Redundant amino acid sequences were removed using CD-hit [22] with a 95% sequence identity cut-off. The protein sequence similarities were assessed by using the BLASTp programs (http://www.ncbi.nlm.nih.gov/BLAST/; until August 15, 2010). Phylogenetic trees were constructed with MEGA 4.0 [45] using the neighbor-joining method [46]. Confidence for tree topologies was estimated by bootstrap values based on 1,000 replicates. Twenty-five representative sequences originating from the rumens or human guts identified by BLASTp analysis were selected and used as references for tree construction.

### Abundance analysis

Gene abundance of each GH family was estimated using distance-based operational taxonomic unit and richness determination (DOTUR) software [23]. Distance matrices of the fragment sequences were calculated at the protein level with the default parameters of protdist in PHYLIP (http://evolution.genetics.washington.edu/phylip.html). Sequences were then assigned to OTUs based on UPGMA (average linkage clustering) implemented in DOTUR with default parameters of precision (0.01) and 1,000 bootstrap replicates.

### Cloning of full-length xylanase genes

Fragments GR67, which showed the phylogenetic novelty (Figure 1) and only appeared once in the GH 10 library (see Table S1), and R8, which represented the most abundant OTU in the GH 11 library (Table S2), were subjected to full-length gene cloning. The flanking regions of these xylanase gene fragments were cloned with eight specific primers for each fragment (Table S6) following the protocol for modified TAIL-PCR [47]. PCR products were electrophoresed on 1.3% agarose gels, cloned into pGEM-T Easy vectors, sequenced, and assembled with the known fragment sequences.

Assembly of the xylanase gene sequences and identification of the open reading frames were performed with programs in Vector NTI 10.3 (InforMax, USA). The signal peptide sequence was predicted with SignalP (http://www.cbs.dtu.dk/services/SignalP/). The DNA and protein sequence identities/similarities were assessed with the BLASTn and BLASTp programs (http://www.ncbi.nlm.nih.gov/BLAST/), respectively.

### Xylanase expression and activity assay

The coding sequences of two representative xylanase genes without the signal peptide were amplified using two primer sets (Table S6), cloned into plasmid pET-22b(+), and transformed into *E. coli* BL21 (DE3) competent cells for recombinant expression. Positive transformants were grown in Luria-Bertani medium containing 100 µg ml^−1^ ampicillin at 37°C to an A_600_ of ∼0.6. Expression was induced with 0.8 mM IPTG at 25°C for 12 h.

Xylanase activity was determined by measuring the release of reducing sugar from substrate with the 3, 5-dinitrosalicylic acid method [48]. Reactions containing 0.1 ml of enzyme preparation and 0.9 ml of 1% (w/v) substrate in McIlvaine buffer (pH 6.0) were incubated at 30°C for 10 min, followed by addition of 1.5 ml of 3, 5-dinitrosalicylic acid reagent. The mixture was boiled for 5 min, cooled to room temperature, and the absorbance at 540 nm (A_540_) was measured. Using a standard curve generated with d-xylose, the absorbance was converted into moles of reducing sugars produced.

### Purification and characterization of recombinant XynGR67 and XynR8

To purify the His-tagged recombinant proteins (XynGR67 and XynR8), culture supernatant was collected after centrifugation (12,000×*g*, 4°C for 15 min) and further concentrated with an ultrafiltration membrane (PES5000; Sartorius Stedim Biotech, Germany). The concentrated supernatant was loaded onto a Ni^2+^-NTA agarose gel column (Qiagen, Germany) with a linear imidazole gradient of 20–200 mM in Tris-HCl buffer (20 mM Tris-HCl, 500 mM NaCl, 10% glycerol, pH 7.6). SDS-PAGE was used to determine the purity and apparent molecular mass of recombinant XynGR67. The protein concentration was determined with the Bradford method [49] with bovine serum albumin as a standard.

The optimal pH for xylanase activity of the purified recombinant XynGR67 and XynR8 were determined at 30°C in buffers of pH 4.0 to 9.0. The enzyme stability at different pHs were estimated by measuring the residual activity after incubating the enzyme solution in buffers at pH 3.0–10.0 at 25°C for 1 h. The buffers used were McIlvaine buffer (0.2 M Na_2_HPO_4_/0.1 M citric acid) for pH 3.0–7.5, and 0.1 M Tris-HCl for pH 7.5–9.0.

The optimal temperature for purified recombinant XynGR67 and XynR8 were determined over the range of 0–80°C in McIlvaine buffer (pH 5.5 for XynR8 and pH 6.0 for XynGR67). Thermostability of XynGR67 was determined after pre-incubating the enzyme in McIlvaine buffer (pH 6.0) at 30°C or 40°C without substrate for various periods while XynR8 was assayed at 55°C and 60°C in McIlvaine buffer (pH 5.5).

The *K*_m_, *V*_max_, and *k*_cat_ values for both recombinant xylanases were determined in McIlvaine buffer (pH 5.5 for XynR8 and pH 6.0 for XynGR67) containing 1–10 mg ml^−1^ birchwood xylan at 40°C and 55°C, respectively. *K*_m_ and *V*_max_ were determined from a Lineweaver-Burk plot with the non-linear regression computer program GraFit (Erithacus Software, UK). Three independent experiments were averaged, and each experiment included three replicates.

The substrate specificity of purified recombinant enzymes were assayed by incubating the enzyme solution with 1% (w/v) substrates including birchwood xylan, beechwood xylan, wheat arabinoxylan or wheat arabinoxylan (insoluble) under standard conditions. Enzymatic activities against *p*-nitrophenyl cellobioside and *p*-nitrophenyl xyloside were examined under standard conditions at a final concentration of 2 mM.

### Nucleotide sequence accession numbers

The nucleotide sequences of GH 10 and 11 xylanase gene fragments were deposited into the GenBank database under accession numbers FJ919156–FJ919224, HM773534–HM773543, and HM773544–HM773578. Accession numbers HQ219689 and HQ219690 were assigned to the full-length xylanase genes *xynR8* and *xynGR67* of the goat rumen contents, respectively.

## Supporting Information

Figure S1 **Phylogenetic analysis based on the partial amino acid sequences of GH 10 xylanase genes detected in the goat and sheep rumen contents and their relationship with the reference sequences retrieved from GenBank.** This tree was constructed using the neighbor-joining method (MEGA 4.0). Sequences from goat rumen were colored in red and those from sheep were in green. Sequence clusters that unique in each rumen were marked with Sheep rumen I–IV or Goat rumen A–C. The lengths of the branches indicate the relative divergence among the amino acid sequences. The reference sequences are marked with a closed diamond (♦) with source strains and GenBank accession numbers in parentheses. The numbers at the nodes indicate bootstrap values based on 1,000 bootstrap replications and bootstrap values (>50) are displayed. The scale bar represents 0.1 amino acid substitutions per position.
(PDF)

Figure S2 **Phylogenetic analysis based on the partial amino acid sequences of GH 11 xylanase genes detected in the goat and sheep rumen contents and their relationship with the reference sequences retrieved from GenBank.** This tree was constructed using the neighbor-joining method (MEGA 4.0). Sequences from goat rumen were colored in red and those from sheep were colored in green. The lengths of the branches indicate the relative divergence among the amino acid sequences. The reference sequences are marked with a closed diamond (♦) with source strains and GenBank accession numbers in parentheses. The numbers at the nodes indicate bootstrap values based on 1,000 bootstrap replications and bootstrap values (>50) are displayed. The scale bar represents 0.1 amino acid substitutions per position.
(PDF)

Table S1 **The GH 10 xylanase gene fragments detected in the goat rumen contents and their closest relative based on amino acid sequence identity and similarity.**
(DOC)

Table S2 **The GH 11 xylanase gene fragments detected in the goat rumen contents and their closest relative based on amino acid sequence identity and similarity.**
(DOC)

Table S3 **The GH 10 xylanase gene fragments detected in the sheep rumen contents and their closest relatives based on amino acid sequence identity and similarity.**
(DOC)

Table S4 **The GH 11 xylanase gene fragments detected in the sheep rumen contents and their closest relatives based on amino acid sequence identity and similarity.**
(DOC)

Table S5 **Summary of the GH 10 and GH 11 xylanase fragment sequences obtained from the goat and sheep rumen contents.**
(DOC)

Table S6 **Primers used for xylanase genes cloning and expression.**
(DOC)
