# Supplementary figures and images for: High Genetic Diversity and Different Distributions of Glycosyl Hydrolase Family 10 and 11 Xylanases in the Goat Rumen
Source: PLoS One. 2011 Feb 3;6(2):e16731. doi: 10.1371/journal.pone.0016731 (PMC3033422; doi:10.1371/journal.pone.0016731)

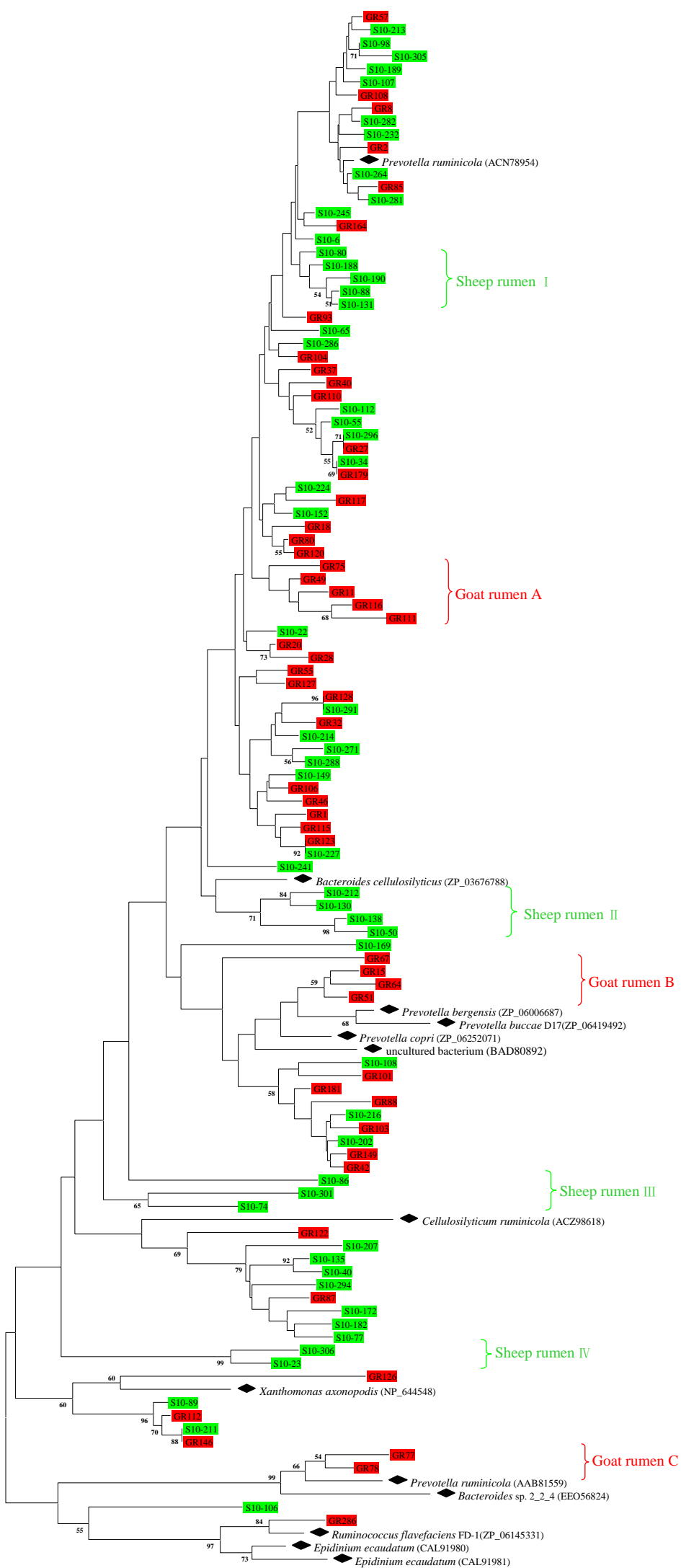

Supplement: Figure S1 — Phylogenetic analysis based on the partial amino acid sequences of GH 10 xylanase genes detected in the goat and sheep rumen contents and their relationship with the reference sequences retrieved from GenBank. This tree was constructed using the neighbor-joining method (MEGA 4.0). Sequences from goat rumen were colored in red and those from sheep were in green. Sequence clusters that unique in each rumen were marked with Sheep rumen I–IV or Goat rumen A–C. The lengths of the branches indicate the relative divergence among the amino acid sequences. The reference sequences are marked with a closed diamond (♦) with source strains and GenBank accession numbers in parentheses. The numbers at the nodes indicate bootstrap values based on 1,000 bootstrap replications and bootstrap values (>50) are displayed. The scale bar represents 0.1 amino acid substitutions per position. (PDF) [file pone.0016731.s001.pdf]

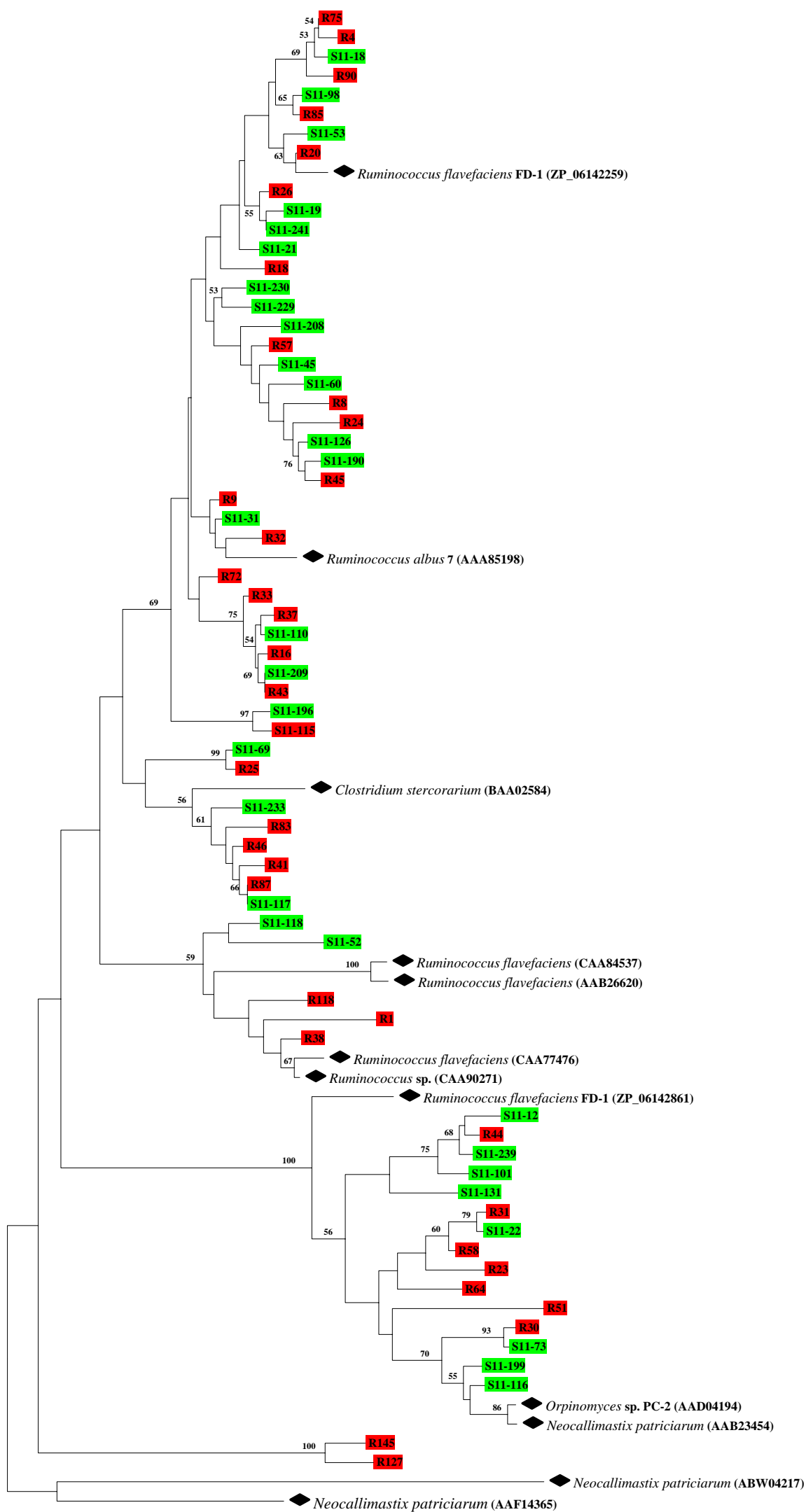

Supplement: Figure S2 — Phylogenetic analysis based on the partial amino acid sequences of GH 11 xylanase genes detected in the goat and sheep rumen contents and their relationship with the reference sequences retrieved from GenBank. This tree was constructed using the neighbor-joining method (MEGA 4.0). Sequences from goat rumen were colored in red and those from sheep were colored in green. The lengths of the branches indicate the relative divergence among the amino acid sequences. The reference sequences are marked with a closed diamond (♦) with source strains and GenBank accession numbers in parentheses. The numbers at the nodes indicate bootstrap values based on 1,000 bootstrap replications and bootstrap values (>50) are displayed. The scale bar represents 0.1 amino acid substitutions per position. (PDF) [file pone.0016731.s002.pdf]
