# Supplementary material for: High Genetic Diversity and Different Distributions of Glycosyl Hydrolase Family 10 and 11 Xylanases in the Goat Rumen
Source: PLoS One. 2011 Feb 3;6(2):e16731. doi: 10.1371/journal.pone.0016731 (PMC3033422; doi:10.1371/journal.pone.0016731)
Supplement: Table S1 — The GH 10 xylanase gene fragments detected in the goat rumen contents and their closest relative based on amino acid sequence identity and similarity. (DOC) [file pone.0016731.s003.doc]

**Table S1. The GH 10 xylanase gene fragments detected in the goat rumen contents and their closest relative based on amino acid sequence identity and similarity*^a^*.**

| GH 10 OTU | Accession no. | Protein size (AA) | Identity/ similarity (%) | No. of sequences | Closest relative (accession No.) |
| --- | --- | --- | --- | --- | --- |
| GR85 | FJ919158 | 92 | 77/80 | 2 | *Prevotella ruminicola* (ACN78954) |
| GR57 | FJ919160 | 93 | 78/85 | 4 | *P. ruminicola* (ACN78954) |
| GR8 | FJ919191 | 88 | 75/83 | 9 | *P. ruminicola* (ACN78954) |
| GR2 | FJ919206 | 92 | 76/79 | 11 | *P. ruminicola* (ACN78954) |
| GR108 | FJ919161 | 97 | 82/91 | 2 | *P. ruminicola* (ACN78954) |
| GR164 | FJ919165 | 93 | 76/79 | 2 | *P. ruminicola* (ACN78954) |
| GR49 | FJ919179 | 92 | 70/75 | 11 | *P. ruminicola* (ACN78954) |
| GR11 | FJ919180 | 92 | 73/75 | 6 | *P. ruminicola* (ACN78954) |
| GR116 | FJ919175 | 92 | 69/75 | 2 | *P. ruminicola* (ACN78954) |
| GR20 | FJ919189 | 92 | 69/75 | 6 | *P. ruminicola* (ACN78954) |
| GR104 | FJ919169 | 92 | 67/74 | 8 | *P. ruminicola* (ACN78954) |
| GR93 | FJ919170 | 92 | 74/78 | 2 | *P. ruminicola* (ACN78954) |
| GR111 | HM773534 | 92 | 67/71 | 2 | *P. ruminicola* (ACN78954) |
| GR80 | FJ919197 | 97 | 71/83 | 6 | *P. ruminicola* (ACN78954) |
| GR75 | FJ919193 | 97 | 69/80 | 2 | *P. ruminicola* (ACN78954) |
| GR120 | FJ919196 | 97 | 69/81 | 3 | *P. ruminicola* (ACN78954) |
| GR127 | FJ919203 | 97 | 60/73 | 4 | *P. ruminicola* (ACN78954) |
| GR18 | FJ919201 | 97 | 67/82 | 3 | *P. ruminicola* (ACN78954) |
| GR117 | FJ919195 | 97 | 62/76 | 30 | *P. ruminicola* (ACN78954) |
| GR28 | FJ919184 | 93 | 64/74 | 3 | *Bacteroides eggerthii* DSM 20697(ZP_03459580)* |
| GR42 | FJ919212 | 84 | 69/87 | 8 | *Prevotella bergensis* DSM 17361 (ZP_06006687) |
| GR103 | FJ919211 | 84 | 69/82 | 1 | *Prevotella buccae* D17 (ZP_06419492) |
| GR64 | FJ919166 | 84 | 68/84 | 1 | *Prevotella buccae* D17 (ZP_06419492) |
| GR55 | FJ919186 | 93 | 64/73 | 8 | *Bacteroides intestinalis* DSM 17393 (ZP_03013017) * |
| GR128 | FJ919183 | 88 | 68/78 | 7 | *B. intestinalis* DSM 17393 (ZP_03013017) * |
| GR115 | FJ919199 | 86 | 64/76 | 1 | *B. intestinalis* DSM 17393 (ZP_03013017) * |
| GR1 | FJ919204 | 86 | 65/73 | 6 | *B. intestinalis* DSM 17393 (ZP_03013017) * |
| GR46 | FJ919207 | 88 | 70/75 | 11 | *B. intestinalis* DSM 17393 (ZP_03013017) * |
| GR27 | HM773535 | 88 | 72/79 | 2 | *B. intestinalis* DSM 17393 (ZP_03013017) * |
| GR240 | HM773536 | 88 | 73/82 | 1 | *B. intestinalis* DSM 17393 (ZP_03013017) * |
| GR32 | HM773541 | 88 | 68/77 | 3 | *B. intestinalis* DSM 17393 (ZP_03013017) * |
| GR10 | FJ919200 | 94 | 70/79 | 1 | *Bacteroides intestinalis* DSM 17393 (ZP_03012528) |
| GR110 | FJ919182 | 88 | 72/81 | 4 | *Bacteroides cellulosilyticus* DSM 14838 (ZP_03678239) * |
| GR123 | HM773538 | 86 | 64/77 | 8 | *B. cellulosilyticus* DSM 14838 (ZP_03678239) * |
| GR106 | FJ919198 | 88 | 68/80 | 1 | *B. cellulosilyticus* DSM 14838 (ZP_03678239) * |
| GR40 | FJ919181 | 88 | 67/77 | 4 | *B. cellulosilyticus* DSM 14838 (ZP_03678239) * |
| GR37 | HM773540 | 86 | 65/76 | 2 | *B. cellulosilyticus* DSM 14838 (ZP_03678239) * |
| GR15 | FJ919216 | 84 | 75/89 | 2 | *Prevotella copri* DSM 18205 (ZP_06252071) |
| GR67 | FJ919221 | 84 | 57/73 | 1 | *P. copri* DSM 18205 (ZP_06252071) |
| GR101 | FJ919213 | 84 | 70/79 | 3 | *P. copri* DSM 18205 (ZP_06252071) |
| GR88 | FJ919215 | 84 | 63/80 | 1 | *P. copri* DSM 18205 (ZP_06252071) |
| GR149 | FJ919178 | 84 | 73/86 | 3 | *P. copri* DSM 18205 (ZP_06252071) |
| GR51 | HM773543 | 84 | 67/85 | 2 | *P. copri* DSM 18205 (ZP_06252071) |
| GR147 | HM773537 | 84 | 71/85 | 24 | *P. copri* DSM 18205 (ZP_06252071) |
| GR126 | FJ919220 | 89 | 55/72 | 1 | *Xanthomonas axonopodis* pv. citri str.306 (NP_644548) |
| GR146 | HM773542 | 87 | 71/80 | 2 | *Verrucomicrobiae bacterium* DG1235 (ZP_05056496) |
| GR112 | FJ919219 | 87 | 72/81 | 3 | *V. bacterium* DG1235 (ZP_05056496) |
| GR77 | FJ919156 | 106 | 69/77 | 1 | *Prevotella ruminicola* (AAB81559) |
| GR78 | FJ919157 | 106 | 68/80 | 1 | *P. ruminicola* (AAB81559) |
| GR122 | FJ919222 | 111 | 38/57 | 2 | *Cellulosilyticum ruminicola* (ACZ98618) |
| GR87 | FJ919223 | 111 | 35/52 | 1 | *C. ruminicola* (ACZ98618) |
| GR286 | HM773539 | 88 | 81/88 | 2 | *Ruminococcus flavefaciens* FD-1 (ZP_06145331) |
| Total 52 |  |  |  | 236 |  |

*^a^* Sequence name was selected to represent each OTU.

* Hypothetical protein.
