# Supplementary material for: High Genetic Diversity and Different Distributions of Glycosyl Hydrolase Family 10 and 11 Xylanases in the Goat Rumen
Source: PLoS One. 2011 Feb 3;6(2):e16731. doi: 10.1371/journal.pone.0016731 (PMC3033422; doi:10.1371/journal.pone.0016731)
Supplement: Table S2 — The GH 11 xylanase gene fragments detected in the goat rumen contents and their closest relative based on amino acid sequence identity and similarity. (DOC) [file pone.0016731.s004.doc]

**Table S2. The GH 11 xylanase gene fragments detected in the goat rumen contents and their closest relative based on amino acid sequence identity and similarity*^a^*.**

| GH 11 OTU | Accession no. | Protein size (AA) | Identity/ similarity (%) | No. of sequences | Closest relative (accession No.) |
| --- | --- | --- | --- | --- | --- |
| R51 | HM773544 | 81 | 66/72 | 2 | *Orpinomyces* sp. FCT 2 (ACY71584) |
| R31 | HM773546 | 76 | 73/80 | 3 | *Neocallimastix frontalis* (ABF66350) |
| R30 | HM773545 | 75 | 82/88 | 5 | *N. frontalis* (ABF66350) |
| R64 | HM773548 | 75 | 73/81 | 1 | *N. frontalis* (ABF66350) |
| R58 | HM773547 | 75 | 73/78 | 2 | *N. frontalis* (ABF66350) |
| R23 | HM773549 | 70 | 80/85 | 1 | *Ruminococcus flavefaciens* FD-1 (ZP_06145331) |
| R44 | HM773550 | 70 | 81/85 | 3 | *R. flavefaciens* FD-1 (ZP_06145331) |
| R38 | HM773551 | 72 | 95/98 | 3 | *Ruminococcus* sp. (CAA90271) |
| R1 | HM773575 | 72 | 81/90 | 10 | *Ruminococcus* sp. (CAA90271) |
| R118 | HM773576 | 72 | 92/94 | 15 | *Ruminococcus* sp. (CAA90271) |
| R145 | HM773577 | 71 | 57/71 | 3 | *Clostridium cellulolyticum* H10 (YP_002505108) |
| R127 | HM773578 | 71 | 56/73 | 2 | *C. cellulolyticum* H10 (YP_0025051 |
| R25 | HM773570 | 71 | 78/88 | 4 | *Anaerocellum thermophilum* DSM 6725 (YP_002572022) |
| R41 | HM773571 | 70 | 82/87 | 6 | *Clostridium stercorarium* (CAD48307) |
| R83 | HM773573 | 70 | 82/90 | 2 | *C. stercorarium* (CAD48307) |
| R46 | HM773574 | 70 | 85/90 | 9 | *C. stercorarium* (CAD48307) |
| R87 | HM773572 | 70 | 85/90 | 5 | *C. stercorarium* (CAD48307) |
| R9 | HM773552 | 71 | 85/91 | 13 | *Ruminococcus albus* 7 (AAA85198) |
| R32 | HM773554 | 71 | 85/92 | 2 | *R. albus* 7 (AAA85198) |
| R72 | HM773553 | 71 | 83/91 | 3 | *R. albus* 7 (AAA85198) |
| R33 | HM773555 | 71 | 83/91 | 1 | *R. albus* 7 (AAA85198) |
| R37 | HM773558 | 71 | 83/92 | 7 | *R. albus* 7 (AAA85198) |
| R16 | HM773556 | 71 | 83/93 | 3 | *R. albus* 7 (AAA85198) |
| R43 | HM773557 | 71 | 84/91 | 5 | *R. albus* 7 (AAA85198) |
| R18 | HM773559 | 71 | 80/87 | 3 | *Ruminococcus flavefaciens* FD-1 (ZP_06142259) |
| R8 | HM773562 | 71 | 87/92 | 15 | *R. flavefaciens* FD-1 (ZP_06142259) |
| R26 | HM773569 | 71 | 85/92 | 3 | *R. flavefaciens* FD-1 (ZP_06142259) |
| R24 | HM773560 | 71 | 85/91 | 5 | *R. flavefaciens* FD-1 (ZP_06142259) |
| R45 | HM773561 | 71 | 84/90 | 2 | *R. flavefaciens* FD-1 (ZP_06142259) |
| R57 | HM773563 | 71 | 81/88 | 1 | *R. flavefaciens* FD-1 (ZP_06142259) |
| R85 | HM773567 | 71 | 91/97 | 7 | *R. flavefaciens* FD-1 (ZP_06142259) |
| R20 | HM773568 | 71 | 95/97 | 3 | *R. flavefaciens* FD-1 (ZP_06142259) |
| R75 | HM773566 | 71 | 89/96 | 2 | *R. flavefaciens* FD-1 (ZP_06142259) |
| R4 | HM773564 | 71 | 88/95 | 6 | *R. flavefaciens* FD-1 (ZP_06142259) |
| R90 | HM773565 | 71 | 88/94 | 5 | *R. flavefaciens* FD-1 (ZP_06142259) |
| Total 35 |  |  |  | 162 |  |

*^a^* Sequence name was selected to represent each OTU.
