# Supplementary material for: High Genetic Diversity and Different Distributions of Glycosyl Hydrolase Family 10 and 11 Xylanases in the Goat Rumen
Source: PLoS One. 2011 Feb 3;6(2):e16731. doi: 10.1371/journal.pone.0016731 (PMC3033422; doi:10.1371/journal.pone.0016731)
Supplement: Table S3 — The GH 10 xylanase gene fragments detected in the sheep rumen contents and their closest relatives based on amino acid sequence identity and similarity. (DOC) [file pone.0016731.s005.doc]

**Table S3. The GH 10 xylanase gene fragments detected in the sheep rumen contents and their closest relatives based on amino acid sequence identity and similarity.**

| OTU *^a^* | Protein size (amino acids) | Identity/ similarity (%) | Amount of sequences | Closest relative (accession No.) |
| --- | --- | --- | --- | --- |
| S10-294 | 111 | 37/58 | 1 | *Cellulosilyticum ruminicola* (ACZ98618) |
| S10-182 | 111 | 38/59 | 1 | *C.ruminicola* (ACZ98618) |
| S10-172 | 111 | 42/62 | 2 | *C.ruminicola* (ACZ98618) |
| S10-77 | 111 | 41/58 | 1 | *C.ruminicola* (ACZ98618) |
| S10-207 | 107 | 37/55 | 1 | *C.ruminicola* (ACZ98618) |
| S10-135 | 111 | 40/56 | 2 | *C.ruminicola* (ACZ98618) |
| S10-40 | 111 | 40/56 | 2 | *C.ruminicola* (ACZ98618) |
| S10-86 | 85 | 50/60 | 1 | *Ruminococcus albus* 8 (ZP_06720104) |
| S10-301 | 84 | 70/82 | 1 | *R. albus* 7 (ZP_07383548) |
| S10-74 | 84 | 63/78 | 2 | *R. albus* 7 (ZP_07383548) |
| S10-106 | 95 | 67/79 | 1 | *Ruminococcus* sp. 18P13 (CBL16579) |
| S10-306 | 97 | 46/65 | 1 | *Clostridium thermocellum* DSM 2360 (ZP_05430148) |
| S10-23 | 97 | 49/66 | 2 | *Clostridium thermocellum* ATCC 27405(YP_001038374) |
| S10-169 | 85 | 55/70 | 1 | *Schizophyllum commune* H4-8 (XP_003027315) |
| S10-216 | 84 | 70/87 | 3 | *Prevotella bergensis* DSM 17361 (ZP_06006687) |
| S10-8 | 84 | 74/88 | 1 | *P. bergensis* DSM 17361 (ZP_06006687) |
| S10-202 | 84 | 74/88 | 2 | *Prevotella copri* DSM 18205 (ZP_06252071) |
| S10-89 | 87 | 73/82 | 1 | *Verrucomicrobiae bacterium* DG1235 (ZP_05056496) |
| S10-211 | 87 | 68/82 | 2 | *V. bacterium* DG1235 (ZP_05056496) |
| S10-212 | 87 | 69/75 | 1 | *Bacteroides eggerthii* DSM 20697 (ZP_03459580)* |
| S10-138 | 90 | 66/76 | 3 | *B. eggerthii* DSM 20697 (ZP_03459580)* |
| S10-241 | 93 | 70/76 | 2 | *B. eggerthii* DSM 20697 (ZP_03459580)* |
| S10-65 | 93 | 67/77 | 5 | *B. eggerthii* DSM 20697 (ZP_03459580)* |
| S10-227 | 86 | 64/77 | 1 | *B. cellulosilyticus* DSM 14838 (ZP_03678239) * |
| S10-130 | 89 | 71/80 | 1 | *Bacteroides intestinalis* DSM 17393 (ZP_03013017)* |
| S10-50 | 90 | 57/70 | 2 | *Bacteroides cellulosilyticus* DSM 14838 (ZP_03676788)* |
| S10-224 | 100 | 72/78 | 2 | *Bacteroides intestinalis* DSM 17393 (ZP_03012528)* |
| S10-152 | 94 | 71/79 | 3 | *B. intestinalis* DSM 17393 (ZP_03012528)* |
| S10-296 | 88 | 72/81 | 40 | *B. intestinalis* DSM 17393 (ZP_03013017)* |
| S10-34 | 88 | 75/81 | 11 | *B. intestinalis* DSM 17393 (ZP_03013017)* |
| S10-14 | 88 | 75/82 | 15 | *B. intestinalis* DSM 17393 (ZP_03013017)* |
| S10-112 | 88 | 75/82 | 2 | *B. intestinalis* DSM 17393 (ZP_03013017)* |
| S10-55 | 88 | 73/81 | 3 | *B. intestinalis* DSM 17393 (ZP_03013017)* |
| S10-149 | 88 | 71/80 | 4 | *B. intestinalis* DSM 17393 (ZP_03013017)* |
| S10-214 | 88 | 72/80 | 2 | *B. intestinalis* DSM 17393 (ZP_03013017)* |
| S10-271 | 88 | 71/82 | 2 | *B. intestinalis* DSM 17393 (ZP_03013017)* |
| S10-288 | 88 | 69/81 | 1 | *B. intestinalis* DSM 17393 (ZP_03013017)* |
| S10-291 | 88 | 68/78 | 4 | *B. intestinalis* DSM 17393 (ZP_03013017) * |
| S10-22 | 94 | 70/79 | 2 | *Prevotella ruminicola* 23 (YP_003575973) |
| S10-286 | 97 | 71/84 | 2 | *P. ruminicola* 23 (YP_003575973) |
| S10-88 | 100 | 74/80 | 17 | *P. ruminicola* 23 (YP_003575973) |
| S10-131 | 100 | 73/82 | 6 | *P. ruminicola* 23 (YP_003575973) |
| S10-80 | 96 | 78/84 | 6 | *P. ruminicola* 23 (YP_003575973) |
| S10-190 | 98 | 75/81 | 5 | *P. ruminicola* 23 (YP_003575973) |
| S10-6 | 95 | 78/81 | 1 | *P. ruminicola* 23 (YP_003575973) |
| S10-245 | 98 | 90/93 | 1 | *P. ruminicola* 23 (YP_003575973) |
| S10-305 | 95 | 76/79 | 21 | *P. ruminicola* 23 (YP_003575973) |
| S10-107 | 98 | 79/84 | 5 | *P. ruminicola* 23 (YP_003575973) |
| S10-232 | 100 | 80/85 | 2 | *P. ruminicola* 23 (YP_003575973) |
| S10-213 | 94 | 75/81 | 8 | *P. ruminicola* 23 (YP_003575973) |
| S10-98 | 98 | 90/95 | 4 | *P. ruminicola* 23 (YP_003575973) |
| S10-281 | 98 | 92/95 | 23 | *P. ruminicola* 23 (YP_003575973) |
| S10-189 | 95 | 77/82 | 8 | *P. ruminicola* 23 (YP_003575973) |
| S10-282 | 96 | 82/89 | 6 | *P. ruminicola* 23 (YP_003575973) |
| S10-264 | 98 | 93/95 | 9 | *P. ruminicola* 23 (YP_003575973) |
| Total 55 |  |  | 258 |  |

*^a^* Sequence name was selected to represent each OTU.

* Hypothetical protein.
