# Supplementary material for: High Genetic Diversity and Different Distributions of Glycosyl Hydrolase Family 10 and 11 Xylanases in the Goat Rumen
Source: PLoS One. 2011 Feb 3;6(2):e16731. doi: 10.1371/journal.pone.0016731 (PMC3033422; doi:10.1371/journal.pone.0016731)
Supplement: Table S4 — The GH 11 xylanase gene fragments detected in the sheep rumen contents and their closest relatives based on amino acid sequence identity and similarity. (DOC) [file pone.0016731.s006.doc]

**Table S4. The GH 11 xylanase gene fragments detected in the sheep rumen contents and their closest relatives based on amino acid sequence identity and similarity.**

| OTU *^a^* | Protein size (amino acids) | Identity/ similarity (%) | Amount of sequences | Closest relative (accession No.) |
| --- | --- | --- | --- | --- |
| S11-22 | 76 | 73/79 | 3 | *Neocallimastix* sp. GMLF2 (ACL80751) |
| S11-73 | 75 | 83/88 | 2 | *Neocallimastix* sp. GMLF2 (ACL80751) |
| S11-116 | 74 | 86/88 | 5 | *Neocallimastix* sp. GMLF2 (ACL80751) |
| S11-199 | 75 | 87/91 | 3 | *Neocallimastix* sp. GMLF2 (ACL80751) |
| S11-239 | 70 | 76/80 | 2 | *Ruminococcus flavefaciens* FD-1 (ZP_06145331) |
| S11-12 | 70 | 82/88 | 3 | *R. flavefaciens* FD-1 (ZP_06145331) |
| S11-101 | 70 | 79/83 | 5 | *R. flavefaciens* FD-1 (ZP_06145331) |
| S11-131 | 70 | 92/96 | 3 | *Ruminococcus flavefaciens* FD-1 (ZP_06142343) |
| S11-60 | 71 | 85/88 | 22 | *Ruminococcus flavefaciens* FD-1 (ZP_06142259) |
| S11-45 | 71 | 85/91 | 2 | *R. flavefaciens* FD-1 (ZP_06142259) |
| S11-208 | 71 | 89/92 | 4 | *R. flavefaciens* FD-1 (ZP_06142259) |
| S11-241 | 71 | 84/91 | 2 | *R. flavefaciens* FD-1 (ZP_06142259) |
| S11-190 | 71 | 85/89 | 4 | *R. flavefaciens* FD-1 (ZP_06142259) |
| S11-126 | 71 | 82/89 | 7 | *R. flavefaciens* FD-1 (ZP_06142259) |
| S11-230 | 70 | 87/88 | 1 | *R. flavefaciens* FD-1 (ZP_06142259) |
| S11-229 | 71 | 85/88 | 16 | *R. flavefaciens* FD-1 (ZP_06142259) |
| S11-21 | 71 | 88/92 | 2 | *R. flavefaciens* FD-1 (ZP_06142259) |
| S11-19 | 70 | 90/96 | 3 | *R. flavefaciens* FD-1 (ZP_06142259) |
| S11-53 | 71 | 92/96 | 1 | *R. flavefaciens* FD-1 (ZP_06142259) |
| S11-98 | 71 | 91/96 | 5 | *R. flavefaciens* FD-1 (ZP_06142259) |
| S11-18 | 71 | 88/96 | 2 | *R. flavefaciens* FD-1 (ZP_06142259) |
| S11-31 | 71 | 88/92 | 11 | *Ruminococcus albus* 7 (ZP_07382492) |
| S11-196 | 70 | 78/84 | 9 | *R. albus* 7 (ZP_07382492) |
| S11-115 | 71 | 79/85 | 4 | *R. albus* 7 (ZP_07382492) |
| S11-209 | 71 | 88/91 | 5 | *Ruminococcus albus* 8 (ZP_06720249) |
| S11-110 | 71 | 88/91 | 5 | *R. albus* 8 (ZP_06720249) |
| S11-117 | 70 | 86/90 | 3 | *Clostridium stercorarium* (CAD48307) |
| S11-233 | 69 | 90/93 | 8 | *C. stercorarium* (CAD48307) |
| S11-69 | 71 | 84/86 | 5 | *Ruminococcus albus* 8 (ZP_06720450) |
| S11-118 | 72 | 95/96 | 7 | *Ruminococcus* sp. ( CAA90271) |
| S11-52 | 72 | 85/96 | 3 | *Ruminococcus* sp. ( CAA90271) |
| Total 31 |  |  | 157 |  |

*^a^* Sequence name was selected to represent each OTU.
