# Supplementary material for: High Genetic Diversity and Different Distributions of Glycosyl Hydrolase Family 10 and 11 Xylanases in the Goat Rumen
Source: PLoS One. 2011 Feb 3;6(2):e16731. doi: 10.1371/journal.pone.0016731 (PMC3033422; doi:10.1371/journal.pone.0016731)
Supplement: Table S5 — Summary of the GH 10 and GH 11 xylanase fragment sequences obtained from the goat and sheep rumen contents. (DOC) [file pone.0016731.s007.doc]

**Table S5.** Statistical analysis of the GH 10 and GH 11 xylanase fragment sequences obtained from the goat and sheep rumen contents.

| **GH family** | **Source** | **Distinct sequences** | **Sequences related to fungi** | **Sequences related to cellulolytic microorganisms** | **Sequences related to noncellulolytic microorganisms** | **Most abundant OTU (sequences)** |
| --- | --- | --- | --- | --- | --- | --- |
| GH 10 | Goat rumen | 52 | 0 | 1 | 51 | GR117 (30) |
|  | Sheep rumen | 55 | 1 | 6 | 48 | S10-296 (40) |
| GH 11 | Goat rumen | 35 | 7 | 30 | 0 | R8 (15) |
|  | Sheep rumen | 31 | 4 | 27 | 0 | S11-60 (22) |
