# Supplementary material for: High Genetic Diversity and Different Distributions of Glycosyl Hydrolase Family 10 and 11 Xylanases in the Goat Rumen
Source: PLoS One. 2011 Feb 3;6(2):e16731. doi: 10.1371/journal.pone.0016731 (PMC3033422; doi:10.1371/journal.pone.0016731)
Supplement: Table S6 — Primers used for xylanase genes cloning and expression. (DOC) [file pone.0016731.s008.doc]

**Table S6. Primers used for xylanase genes cloning and expression.**

| Primer | Sequence (5'→3') *^a^* | Size (bp) |
| --- | --- | --- |
| GR67-uSP1 | CGATCCTGATTCCTTTCGCCTTCAGCTTCCTG | 32 |
| GR67-uSP2 | GCCTTCAGCTTCCTGGCGTATTCGATCACC | 30 |
| GR67-uSP3 | GAGTTCCACATGTTGAAATCGTTGTAGTAGAGTTCGGC | 38 |
| GR67-uSP4 | CTGAGAATTGTGGATGTTGCCCTCGTCATCGAAC | 34 |
| GR67-dSP1 | GTTCGATGACGAGGGCAACATCCACAATTCTCAG | 34 |
| GR67-dSP2 | CGATGACGAGGGCAACATCCACAATTCTCAGTG | 33 |
| GR67-dSP3 | CGCGAAGGAATAGGACCCGATTTCATCGAACTG | 33 |
| GR67-dSP4 | GCCGAACTCTACTACAACGATTTCAACATGTGGAACTC | 38 |
| R8-uSP1 | GGTGCCGTCGATGGATGGCTGTTCAAC | 27 |
| R8-uSP2 | GTCGATTGAAGGCTGTTCAACGCGTGTG | 28 |
| R8-uSP3 | GCGGGTGGTTTTGTATATGTCATAGACTGCAC | 32 |
| R8-uSP4 | CACAATGTAGAATTCCACGAGCGGAGATTTGGTC | 34 |
| R8-dSP1 | GACCAAATCTCCGCTCGTGGAATTCTACATTGTG | 34 |
| R8-dSP2 | CCGCTCGTGGAATTCTACATTGTGGAATCATGG | 33 |
| R8-dSP3 | CACCATCTGCCATCGGCAATGTCACCGTG | 29 |
| R8-dSP4 | CACCCGCGTTGAACAGCCATCCATCGAC | 28 |
| GR67-m-F | GCC**GAATTC**TGCAGGGCTCAAGGACGACTACGCCGGCAAG | 40 |
| GR67-m-R | GGA**GCGGCCGC**CTTCCTTGGTTTTTGCGAAGTCTTAACGTCTC | 43 |
| R8-m-F | GAA**CCATGGAT**ATGGCGGCAATGCTTGCAGGCTGCGGAAATAGTG | 45 |
| R8-m-R | GGA**GCGGCCGC**CTCATTGACATTAACTTCAATGTCGGTCGCTTCAG | 49 |

*^a^* Restriction sites used for expression are underlined.
